# Editorial: Smoking and Schizophrenia

**DOI:** 10.3389/fpsyt.2019.00738

**Published:** 2019-10-17

**Authors:** David Castle, Amanda L. Baker, Billie Bonevski

**Affiliations:** ^1^Department of Psychiatry, The University of Melbourne, Melbourne, VIC, Australia; ^2^Mental Health Research Unit, St Vincent’s Hospital, Melbourne, VIC, Australia; ^3^School of Medicine and Public Health, University of Newcastle, Callaghan, NSW, Australia

**Keywords:** smoking, schizophrenia, cessation, nicotine, physical health

The association between cigarette smoking and schizophrenia is well established. Rates of smoking among people with schizophrenia are markedly higher than in the general population. In many countries where public health campaigns have reduced the overall prevalence of smoking amongst the general population, there has been little impact on rates of smoking amongst people with schizophrenia. The series of articles in this collection aims to understand the reason for the high smoking rates amongst people with schizophrenia, as well as to explore both barriers and facilitators towards smoking cessation in this vulnerable group.

The reason why people with schizophrenia do smoke at such high rates is complicated and include psychosocial factors, milieu issues such as initiation of smoking on inpatient wards (albeit many have now banned smoking) as well as social affiliation. However, there is more to it than this and the effects of nicotine on the symptoms of schizophrenia as well as cognition need to be factored into this understanding. The paper by Lucatch et al. tackles the topic of neurobiological underpinnings of tobacco smoking in people with schizophrenia, with an overview of how nicotine may serve to ameliorate some of the perturbations in dopaminergic, glutamatergic, and GABAergic pathways. These authors conclude that an understanding of these neurobiological parameters is important in treatment paradigms where the understandings can be integrated into psychosocial interventions. A case control study presented by Stramecki et al. explores the association between cigarette smoking and cognitive function in people with schizophrenia and controls without schizophrenia; roughly half in each group were cigarette smokers. A comprehensive neuropsychological battery was administered, with results suggesting that cigarette smoking is associated with impairment in delayed memory in people with schizophrenia. The authors noted that longitudinal studies are required to establish causal associations.

Scott et al. address a very interesting issue of whether smoking is a cumulative causal factor for schizophrenia and related disorders. They provide an overview of existing studies that have addressed this issue and conclude that despite methodological shortcomings (including failure to adjust for certain confounders such as childhood trauma or prenatal tobacco exposure), there is “substantial though inconclusive evidence supporting a causal relationship between tobacco smoking and an increased risk of schizophrenia spectrum disorder.” The potential public health implications of this finding are profound.

The bulk of the rest of the articles in this collection address the issue of smoking cessation and how to help smokers with schizophrenia to quit. Lum et al. performed a comprehensive systematic review of the literature on psychosocial barriers and facilitators to smoking cessation in people with schizophrenia. They included 23 articles: 20 were quantitative and 3 qualitative. In terms of barriers to smoking cessation, cravings and addiction were the most strongly endorsed, followed by the perception that negative symptoms worsened when quitting smoking. The review also showed that people with schizophrenia believed that smoking helps them manage stress and maintain social relationships; health concerns were seen as reasons to quit smoking. These important findings are echoed and expanded by Cocks et al. who bring a “lived experience lens” to this particular issue. The authors suggest that a recovery orientated approach could integrate treatments that have an evidence base in terms of smoking cessation.

A somewhat more challenging view is provided by Twyman et al. in a qualitative study of community mental health staff and consumers about the role of tobacco in their lives as well as the impact of these issues on smoking cessation. Themes identified by staff included some degree of fatalism in that they saw people continuing to smoke as their choice rather than an addiction and identified a tension between offering smoking cessation programs and “free choice.” Consumers saw smoking as part of their life and social networks and as a way of “maintaining control.” Social support to quit was an important theme. These authors conclude that education and training for staff within community mental health services is imperative.

In terms of specific programs addressing smoking cessation, Curtis et al. assessed the uptake and impact of a smoking cessation program in young people with a psychotic illness. Of 61 young people who were eligible for the program, 41 (67%) engaged in the program; a third of these had the full intervention and further third received only a brief intervention. Nearly half of those receiving the full intervention and a quarter of those receiving the brief intervention dropped out; 28% of those completing the full intervention achieved smoking cessation. The authors emphasised the potential for impacting smoking behaviours in youth in the early phases of severe mental illness.

Baker et al. outline a current randomized controlled trial with smokers with a severe mental illness. A brief peer delivered intervention around smoking is being compared to an intervention that includes the same brief intervention with proactive referral to a tailored cognitive behavioural intervention offered by “Quitline,” along with nicotine replacement therapy. This ambitious study aims to recruit nearly 400 smokers over 3 years. The trial asks an important question as to whether a “minimal intervention” can address smoking amongst people with schizophrenia. In such trials, it is important also to have an assessment of cost, and a companion paper by Sweeney et al. provides the protocol for the economic evaluation of this Quitlink program.

Seeking to strengthen future research efforts, the editors and other international experts (Baker et al.) propose research priorities. These are i) understanding more about the associations between smoking, smoking cessation, and symptomatology; ii) targeted, adaptive, and responsive behavioural interventions evaluated by smarter methodologies; and iii) improvements in delivery of interventions, especially within health care settings. A collaborative international research agenda, with partnerships between bodies overseeing mental health treatment and smoking cessation, would likely add momentum to research efforts. Using existing structures to support an international collaborative effort between national psychiatry, mental health, and tobacco control research societies is likely to provide a solid platform for research growth in this area. These societies can connect researchers and break down research siloes. Strategic and targeted funding from international and national organisations focussed on supporting innovation in treatment for smokers with schizophrenia is critical. Finally, it is imperative that we grow our research capacity in this field, bringing together academic researchers and clinicians to ensure research innovations are translated into policy and practice change.

## Author Contributions

DC, AB and BB all collaborated in the construction of the editorial, shared the writing between them, and all approved the final version.

## Conflict of Interest

The authors declare that the research was conducted in the absence of any commercial or financial relationships that could be constructed as a potential conflict of interest.

